# Registration of Functioning of a Single Horseradish Peroxidase Macromolecule with a Solid-State Nanopore

**DOI:** 10.3390/ijms242115636

**Published:** 2023-10-27

**Authors:** Yuri D. Ivanov, Alexander N. Ableev, Ivan D. Shumov, Irina A. Ivanova, Nikita V. Vaulin, Denis V. Lebedev, Anton S. Bukatin, Ivan S. Mukhin, Alexander I. Archakov

**Affiliations:** 1 Institute of Biomedical Chemistry, 10, Pogodinskaya St., Moscow 119121, Russia; ableev@mail.ru (A.N.A.); shum230988@mail.ru (I.D.S.); i.a.ivanova@bk.ru (I.A.I.); alexander.archakov@ibmc.msk.ru (A.I.A.); 2 Laboratory of Renewable Energy Sources, St. Petersburg Academic University, 8/3, Khlopina st., St. Petersburg 194021, Russia; nikitavaylin@mail.ru (N.V.V.); denis.v.lebedev@gmail.com (D.V.L.); antbuk.fiztek@gmail.com (A.S.B.); imukhin@yandex.ru (I.S.M.); 3 Institute for Analytical Instrumentation RAS, 31-33 Lit. A, Ivana Chernykh St., St. Petersburg 198095, Russia; 4 Institute of Chemistry, Saint Petersburg State University, 7/9, Universitetskaya Nab., St. Petersburg 199034, Russia; 5 Higher School of Engineering Physics, Peter the Great Polytechnic University, 26, Polytehnicheskaya St., St. Petersburg 194021, Russia

**Keywords:** nanopore detector, solid-state nanopore, horseradish peroxidase, enzymatic activity

## Abstract

Currently, nanopore-based technology for the determination of the functional activity of single enzyme molecules continues its development. The use of natural nanopores for studying single enzyme molecules is known. At that, the approach utilizing artificial solid-state nanopores is also promising but still understudied. Herein, we demonstrate the use of a nanotechnology-based approach for the investigation of the enzymatic activity of a single molecule of horseradish peroxidase with a solid-state nanopore. The artificial 5 nm solid-state nanopore has been formed in a 40 nm thick silicon nitride structure. A single molecule of HRP has been entrapped into the nanopore. The activity of the horseradish peroxidase (HRP) enzyme molecule inserted in the nanopore has been monitored by recording the time dependence of the ion current through the nanopore in the course of the reaction of 2,2′-azino-bis(3-ethylbenzothiazoline-6-sulfonate) (ABTS) oxidation reaction. We have found that in the process of ABTS oxidation in the presence of 2.5 mM hydrogen peroxide, individual HRP enzyme molecules are able to retain activity for approximately 700 s before a decrease in the ion current through the nanopore, which can be explained by structural changes of the enzyme.

## 1. Introduction

Nanotechnology-based approaches are becoming widely utilized in single-molecule studies of enzymes. These approaches include atomic force microscopy (AFM), which is commonly employed for estimating the activity of single enzyme molecules. For instance, in [1], the semi-contact mode of AFM was used for the monitoring of height fluctuations of lysozyme molecules adsorbed on mica. The time dependencies of the height fluctuations were obtained without scanning. The AFM tip was positioned above the lysozyme monolayer under various conditions: in buffer, in the presence of the lysozyme substrate, and in the presence of its inhibitor. In the presence of the oligosaccharide substrate, the height fluctuations with an amplitude of 1 nm and a period of 50 ms were observed. In contrast, in the presence of the inhibitor, the amplitude of the fluctuations reduced to the level observed before the substrate addition. The interpretation of these findings suggests that the height fluctuations of lysozyme correspond to its conformational changes during the hydrolysis reaction. Furthermore, these fluctuations can be influenced by differences in height and elasticity of the lysozyme-substrate transition complex. A similar approach was employed in other studies [2,3]. Therein, the fluctuations of IgG antibodies and the functioning of the chitosanase enzyme were investigated. These examples illustrate how AFM allows one to monitor enzymatic activity by tracking the oscillation of the AFM probe. Furthermore, an analogous approach was employed in order to measure the activity of single molecules of cytochrome P450 BM3 enzyme [4]. 

With respect to AFM, one should emphasize that the implementation of this approach requires expensive equipment. In addition, the AFM experiments are time-consuming. An alternative nanotechnology-based approach to the registration of the functioning of single enzyme molecules is based on the use of nanopores. In numerous papers, the use of natural nanopores was reported [5,6,7,8,9,10]. The approach employing natural nanopores has certain limitations, which are connected with the size of these nanopores. This is why the use of natural nanopores for studying a wide range of enzymes is still limited. 

In contrast to the use of natural nanopores, the application of artificial solid-state nanopores overcomes the above-discussed limitation since their diameters can be varied upon the nanopore fabrication. Accordingly, their use allows one to study diverse enzymes. According to the literature, the sizes of protein molecules vary from several nanometers [11] to one micron [12]. At that, the size of many heme-containing enzyme proteins, such as horseradish peroxidase [13] and cytochromes P-450 [14], is about 5 nm. These enzyme proteins are of particular interest. Thus, the technology based on solid-state nanopores with a pore diameter of about 5 nm is the most promising nanopore-based approach for investigating enzyme activity [15]. These solid-state nanopores can be fabricated by different methods, including focused ion beam (FIB) [15], controlled dielectric breakdown (SDB) [16,17], and electron beam drilling (EBD) [18]. The material commonly used for the fabrication of solid-state nanopores is silicon nitride (Si_x_N_y_, hereafter abbreviated SiN). 

Enzyme systems play key roles in diverse metabolic processes in living organisms [19]. Herein, the horseradish peroxidase (HRP) enzyme, which pertains to a broad class of peroxidases, has been selected as an object owing to its well-known properties. HRP participates in catalytic oxidation reactions involving a wide range of organic and inorganic compounds in the presence of hydrogen peroxide [20]. The molecular weight of HRP is approximately 40 kDa [21]. The catalytic activity of HRP can be assessed using a distinctive reaction involving its substrate, 2,2′-azino-bis(3-ethylbenzothiazoline-6-sulfonate) (ABTS), and hydrogen peroxide, described by Sanders et al. [22].

In our present research, the EBD technology has been employed in order to fabricate the solid-state nanopore in SiN. The HRP enzyme was inserted into the nanopore. After that, the ABTS oxidation reaction was started by adding hydrogen peroxide (H_2_O_2_). The change in the current flowing through the nanopore during the enzyme functioning was monitored.

## 2. Results

### 2.1. Nanopore Fabrication

**Figure 1:**
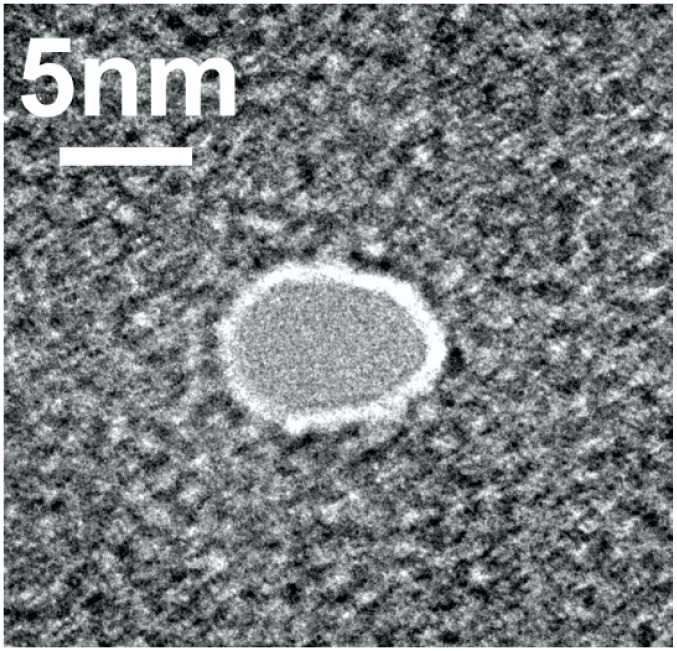
Typical TEM image of a nanopore formed by EBD.

According to X-ray data, the dimensions of horseradish peroxidase (HRP) were determined to be approximately 4.3 × 4.8 × 5.8 nm [13]. The nanopore with a diameter of 5 nm was fabricated in 40 nm thick SiN. Figure 1 displays a typical transmission electron microscopy (TEM) image of the so-fabricated nanopore.

For the pore, whose image is displayed in Figure 1, the efficient diameter and length were *d* = 5 nm and *l* = 40 nm, respectively. The nanopore was observed to have a size slightly smaller than the maximum size of HRP (5.8 nm) but larger than its minimum size (4.3 nm). Accordingly, the nanopore was suitable for accommodating the HRP molecule, preventing it from passing through while allowing for its incorporation within the nanopore.

### 2.2. Testing of Nanopore Performance

For the registration of the HRP enzymatic activity with the nanopore-based detector, tests were performed in order to assess the successful incorporation of HRP into the nanopore. Prior to these tests, the chip was washed with ultrapure water. Subsequently, both chambers of the measuring cell were filled with 2 mM PBS-D buffer.

Control experiments were conducted in order to obtain the time dependence of the ion current through the nanopore at a voltage of −200 mV. Pure buffer was initially added to the cis chamber, followed by the addition of ABTS at a concentration of 0.3 mM. Half of the solution volume was then evacuated from the cis chamber, and 0.003% H_2_O_2_ solution was added. In this experiment, we observed no significant changes in the current signal fluctuations, which remained below 50 pA.

### 2.3. Nanopore Experiments

**Figure 2:**
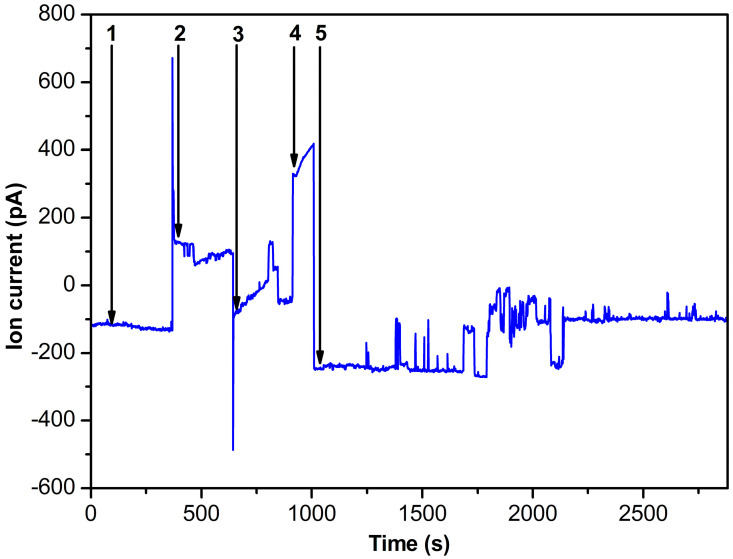
Time dependence of the ion current through the nanopore. Arrows indicate (1)—addition of 10^−8^ M HRP, voltage U = −200 mV; (2)—voltage polarity switching to U = 200 mV; (3)—voltage polarity switching to U = −200 mV; (4)—addition of 0.3 mM ABTS, voltage U = −200 mV; (5)—addition of H_2_O_2_, voltage U = −200 mV.

For the experiments involving HRP incorporation, a buffer solution containing HRP at a concentration of 10^−8^ M was introduced into the cis-chamber of the measuring cell, while the trans-chamber was filled with HRP-free buffer solution. Figure 2 illustrates the ion current dependence over time during the addition of the corresponding solution to the cis part of the measuring cell.

The curve shown in Figure 2 indicates that at 10^−8^ M HRP concentration and U = −200 mV voltage applied to the cell (cis-chamber/trans-chamber), the ion current through the nanopore was approximately −100 pA. It should be noted that at the pH of the buffer solution used, the HRP molecule bears a negative charge. The latter directs the enzyme into the nanopore under the applied voltage conditions. The voltage polarity was then switched to U = 200 mV (cis-chamber/trans-chamber), resulting in a change in the current flowing through the nanopore. At that, the current reached approximately 100 pA. Then, the cell polarity was switched again to U = −200 mV (cis-chamber/trans-chamber), forcing the nanopore blockade. At that, the ion current decreased to zero. The cell was subsequently filled with ABTS solution. After the subsequent evacuation of half of the ABTS solution volume, H_2_O_2_ was added in order to initiate the ABTS oxidation reaction. This led to a series of significant signal fluctuations, which persisted for about 1800 s, indicating the enzyme functioning.

In the spectrophotometry experiments, we observed that under the conditions described in Section 4.4, the absorbance of the solution containing 1 nM HRP reached the value of 0.4 after five minutes of the ABTS oxidation reaction. This indicated that the enzyme is able to catalyze the ABTS oxidation in 2 mM PBS-D buffer, i.e., is active. No change in the solution absorbance was observed in blank spectrophotometry experiments performed in the absence of the enzyme.

## 3. Discussion

The diameter of the nanopores to be fabricated depends on the aim of the study. For instance, for DNA sequencing, 4 nm diameter nanopores are typically employed [23]. Through such pores, translocation of a DNA strand (whose diameter is about 2.4 nm) is possible. For capturing and retention of biological macromolecules, it is reasonable to use nanopores, whose diameter is somewhat smaller than the size of the macromolecules to be captured. This consideration determined the use of 5 nm nanopores in our experiments.

Our study demonstrates the successful use of a 5 nm SiN solid-state nanopore for monitoring the enzymatic activity of a single HRP molecule. The latter was inserted into the nanopore. In our experiments, the time dependence of the ion current through the nanopore during the enzyme catalytic cycle was recorded. During the catalytic cycle, current pulses were observed, indicating changes in the nanopore size. The latter can be attributed to alterations in the shape of the enzyme molecule confined within the nanopore. These current fluctuations persisted for a considerably long (1800 s) time. After this, the enzyme was found to be still active. At that, after 1700 s (i.e., approximately 700 s after the addition of H_2_O_2_), a decrease in the ion current was observed. This decrease can be explained by structural changes in the enzyme, leading to a substantial nanopore blockade and thus hindering the ion flow and decreasing the current. These findings demonstrate that individual HRP enzyme molecules are able to retain activity for approximately 700 s before undergoing structural changes, which decrease the nanopore conductivity. Blank experiments were carried out without the enzyme. In the blank experiments, we observed no significant current fluctuations in the presence of ABTS and H_2_O_2_ in the measuring cell. These results hold significant implications for comprehending enzyme functionality and enable the analysis of enzyme behavior at the single-molecule level. In the future, we intend to perform experiments with a number of HRP molecules in order to obtain the enzymatic activity distribution of these molecules.

## 4. Materials and Methods

### 4.1. Chemicals and Enzyme

The enzyme (peroxidase from horseradish; Cat. #6782) and its substrate ABTS were purchased from Sigma (St. Louis, MO, USA). The experiments were performed in 2 mM Dulbecco’s modified phosphate buffered saline (hereafter PBS-D buffer) with a pH of 7.4. Ultrapure deionized water, obtained with a Simplicity UV system (Millipore, Molsheim, France), was used in all experiments. The HRP enzyme was purchased from Sigma (St. Louis, MO, USA). The solution containing 0.3 mM ABTS was prepared by dissolving 8.2 mg of ABTS in 50 mL of 2 mM PBS-D immediately prior to the experiments. Hydrogen peroxide (H_2_O_2_) solution was diluted with 2 mM PBS-D to a final concentration in the cell of 0.003% (*v*/*v*).

### 4.2. Nanopore Fabrication and Characterization

It should be emphasized that the nanopore diameter can be monitored in situ directly inside the transmission electron microscope in the course of the nanopore formation. The nanopore was formed by EBD in a SiN chip. Its diameter was approximately 5 nm, and its thickness was ~40 nm. This nanopore was obtained with a JEM 2100F high-resolution transmission electron microscope.

In our study, a two-step approach to the characterization of single solid-state nanopores was employed. In the first step, the shape and size of the nanopores were analyzed by TEM directly after their formation. Typical TEM images of the nanopores fabricated are displayed in Figure S1 (see Supplementary Information File). At that, nanopores with diameters of >5 nm were not used in further experiments. Figure S2 displays a histogram of the pore diameter distribution. From this histogram, one can see that the majority of the fabricated nanopores had a diameter of about 5 nm (see Supplementary Information File).

In the second step, we performed an analysis of the current-voltage characteristics (CVCs) obtained in a potentiostatic mode. The CVCs were recorded in the following way. A chip containing a single nanopore was positioned between two chambers of the measuring cell. In this way, the pore divided the cell into two independent volumes. A Ag/AgCl electrode was immersed into each volume. This system was filled with 1 M aqueous KCl solution. Then, a constant voltage was applied to the electrodes for 800 s. At that, an ionic current flowing through the nanopore was recorded. In this way, a dependence of the ionic current on the applied voltage (CVC) was obtained for each nanopore studied. For nanopores of cylindrical shape with a constant surface charge density (with a non-conductive surface), CVC is known to be linear [24,25,26]. Accordingly, in our experiments reported, only the nanopores with linear CVCs were employed. Typical CVC obtained for a 5 nm solid-state nanopore is shown in Figure S3, while Figure S4 displays the CVCs calculated for nanopores of various lengths and diameters according to Kowalczyk et al. [27] (see Supplementary Information File).

### 4.3. Nanopore-Based Detector

The nanopore-based detector comprised the measuring cell, which was divided into two chambers with the SiN nanopore integrated into the partition. That is, the chip with the nanopore served as the partition between the two chambers (cis- and trans-). The cell was washed with ultrapure water immediately prior to the measurements.

### 4.4. Electrical Measurements

Both chambers (cis- and trans-) were filled with 700 μL of 2 mM PBS-D (pH 7.4). In the measurements, a voltage was applied to both chambers, and the current flowing through the nanopore was recorded. Ag/AgCl electrodes were immersed into the cis- and trans-chambers in order to apply the electric voltage for recording the ion current through the nanopore. The detector was shielded with a Faraday cell. The current flowing through the nanopore was measured with a patch-clamp amplifier with an intrinsic noise level of 0.3 fA in a frequency band of 1000 Hz. The voltage varied within the range from −300 to 300 mV. The current signal from the nanopore was recorded at a frequency of 10 kHz using a 16-bit analog-to-digital converter (ADC). This signal was then filtered through a low-pass Butterworth filter with a frequency of 1000 Hz. After the measurement cycle, the SiN chip was washed with ultrapure water in order to eliminate salt residues and reinstalled into the measuring cell.

### 4.5. Spectrophotometry

Spectrophotometry measurements were performed according to the modified technique reported by Sanders et al. [22]. In the originally reported technique, the measurements are performed in phosphate-citrate buffer [22]. According to Drozd et al. [28], phosphate buffers can also be successfully employed, but the pH should be acidic (5.0) in order to achieve optimal performance [29].

Accordingly, our spectrophotometry measurements were aimed at finding out whether HRP is able to catalyze the oxidation of ABTS by H_2_O_2_ in 2 mM PBS-D buffer employed in the nanopore-based detector. That is, the modification of the technique consisted of the use of the PBS-D buffer instead of the phosphate-citrate one. Other experimental conditions were similar to those recommended by Sanders et al. [22]. Namely, the concentrations of the enzyme, ABTS and H_2_O_2_ were 1 nM, 0.3 mM, and 2.5 mM, respectively, and the absorbance of the solution was recorded at 405 nm wavelength in a 1 cm long quartz cell. An Agilent 8453 spectrophotometer was used in the measurements. The time dependencies of the absorption were recorded for five minutes in at least three technical replicates.

## 5. Conclusions

A solid-state SiN nanopore with a diameter of 5 nm has been fabricated. A single molecule of HRP enzyme has been captured within this nanopore. The functioning of the enzyme in the course of catalysis of ABTS oxidation by hydrogen peroxide has been monitored by recording the time dependence of ion current through the nanopore. Importantly, 1800 s exhibited observable current fluctuations during enzyme functioning, which were notably absent in the absence of ABTS and H_2_O_2_. Accordingly, the 5 nm solid-state nanopore can be successfully used for monitoring the enzymatic activity of a single HRP molecule. These findings provide valuable insights into the fundamental mechanisms governing enzyme function at the single-molecule level.

## Data Availability

Correspondence and requests for materials should be addressed to Y.D.I.

## Supplementary Materials

The following supporting information can be downloaded at https://www.mdpi.com/article/10.3390/ijms242115636/s1**.**
