# Supplementary material for: Registration of Functioning of a Single Horseradish Peroxidase Macromolecule with a Solid-State Nanopore
Source: Int J Mol Sci. 2023 Oct 27;24(21):15636. doi: 10.3390/ijms242115636 (PMC10647385; doi:10.3390/ijms242115636)
Supplement: Supplementary file 1 [file ijms-24-15636-s001.zip › ijms-2587181-SI.pdf]

Supplementary Information to:

Registration of Functioning of a Single Horseradish Peroxidase Macromolecule with a Solid-State Nanopore

Yuri D. Ivanov [1,*], Alexander N. Ableev [1], Ivan D. Shumov [1], Irina A. Ivanova [1], Nikita V. Vaulin [2,3], Denis V. Lebedev [2,3,4], Anton S. Bukatin [2,3], Ivan S. Mukhin [2,5] and Alexander I. Archakov [1]

[1] Institute of Biomedical Chemistry, 10, Pogodinskaya St., Moscow 119121, Russia; ableev@mail.ru (A.N.A.); shum230988@mail.ru (I.D.S.); i.a.ivanova@bk.ru (I.A.I.); alexander.archakov@ibmc.msk.ru (A.I.A.)

[2] Laboratory of Renewable Energy Sources, St. Petersburg Academic University, 8/3, Khlopina st., St. Petersburg 194021, Russia; nikitavaylin@mail.ru (N.V.V.); denis.v.lebedev@gmail.com (D.V.L.); antbuk.fiztek@gmail.com (A.S.B.); imukhin@yandex.ru (I.S.M.)

[3] Institute for Analytical Instrumentation RAS, 31-33 Lit. A, Ivana Chernykh St., St. Petersburg 198095, Russia

[4] Institute of Chemistry, Saint Petersburg State University, 7/9, Universitetskaya Nab., St. Petersburg 199034, Russia

[5] Higher School of Engineering Physics, Peter the Great Polytechnic University, 26, Polytehnicheskaya St., St. Petersburg 194021, Russia

* Correspondence: yurii.ivanov.nata@gmail.com

The nanopores were fabricated by EBD in a SiN chip. Figure S1 displays typical TEM images of different solid-state nanopores fabricated.

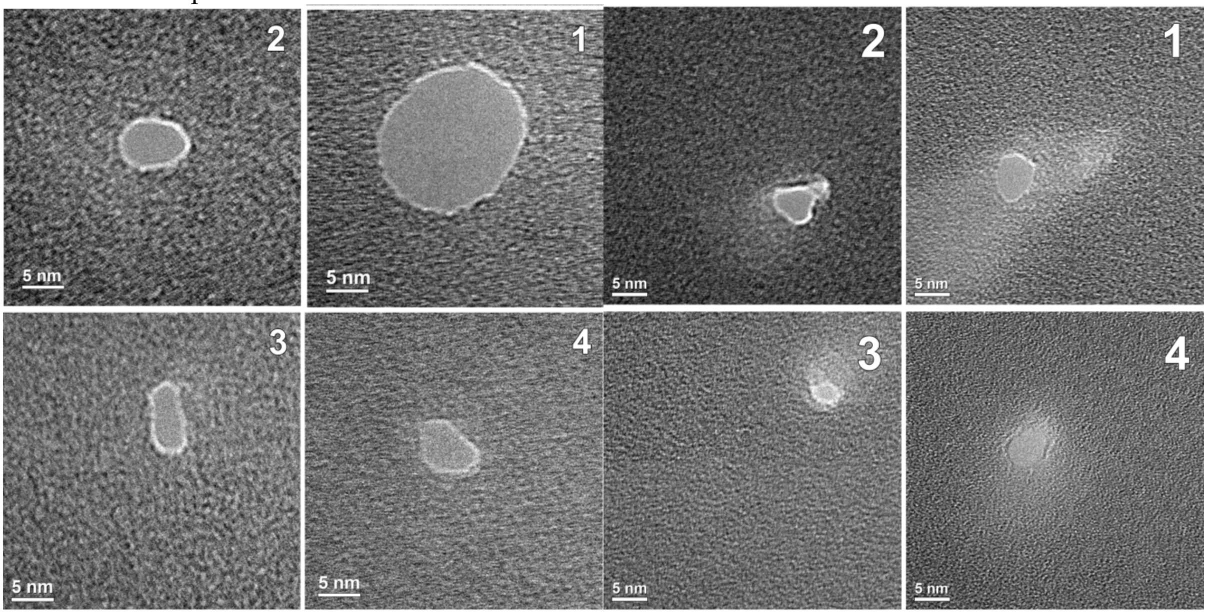

Figure S1. Typical TEM images of different solid-state nanopores.

Figure S2 displays a histogram of the pore diameter distribution. From this histogram one can see that the majority of the fabricated nanopores had a diameter of about 5 nm (see Supplementary Information file).

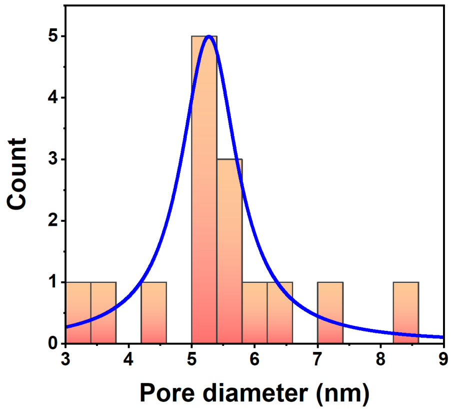

Figure S2. Typical TEM images of different solid-state nanopores.

In order to additionally characterize the shape and size of the solid-state nanopores fabricated, we performed analysis of the current-voltage characteristics (CVCs) obtained in potentiostatic mode as described in section 4.2. Figure S3 displays typical CVC obtained for a 5 nm solid-state nanopore.

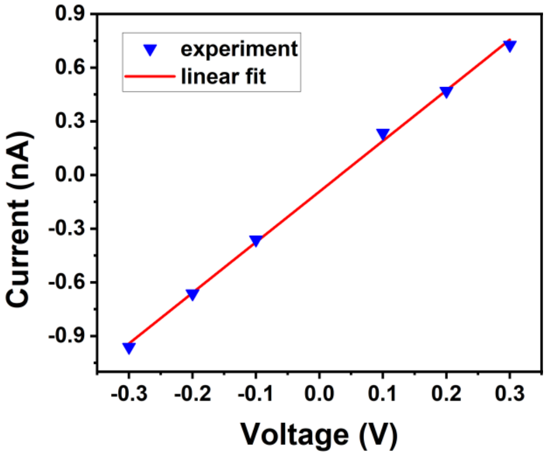

Figure S3. Typical current-voltage characteristic (CVC) of a 5 nm solid-state nanopore obtained in potentiostatic mode in 1 M KCl.

For nanopores of cylindrical shape with a constant surface charge density (with a non-conductive surface), CVC is known to be linear [1-3]. In our experiments reported, only the nanopores with linear CVCs were employed. Furthermore, the efficient diameter *d* of a nanopore can also be determined based on the slope of its CVC [4]:

$$G = \sigma\left(\frac{4l}{\pi d^2} + \frac{1}{d}\right)^{-1},$$

where *G* is the ionic conductance, *l* is the length of the nanopore, and *σ* is the specific electrical conductivity of the electrolyte solution [4].

Thus, the size of a nanopore considerably influences its conductivity and the respective CVCs. Figure S4 displays the CVCs calculated for nanopores of various *l* and *d*.

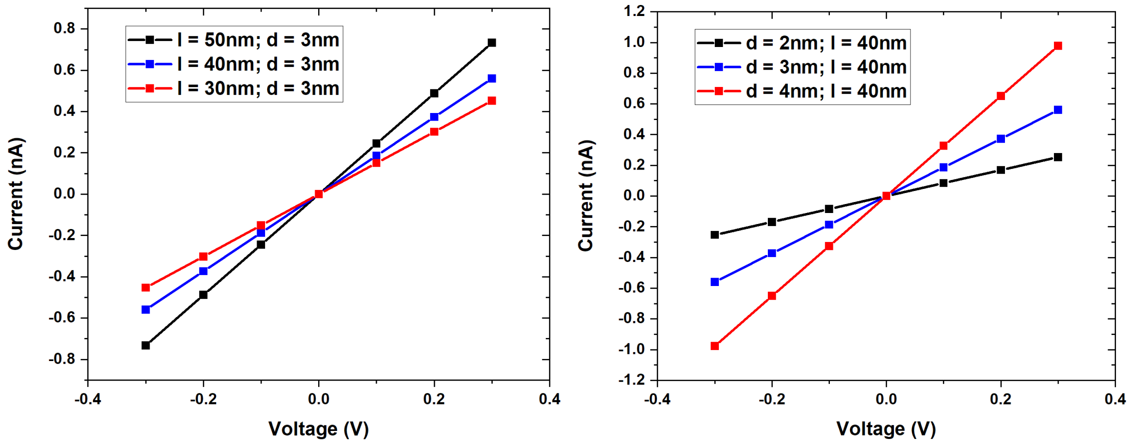

Figure S4. CVCs calculated for nanopores of various *l* and *d*.

In this way, the nanopores used in our experiments were comprehensively characterized.

References

1. Esfandiar, A.; Radha, B.; Wang, F.C.; Yang, Q.; Hu, S.; Garaj, S.; Nair, R.R.; Geim, A.K.; Gopinadhan, K. Size effect in ion transport through angstrom-scale slits. *Science* **2017**, *358*, 511-513. https://doi.org/10.1126/science.aan5275.

2. Yazda, K.; Tahir, S.; Michel, T.; Loubet, B.; Manghi, M.; Bentin, J.; Picaud, F.; Palmeri, J.; Henn, F.; Jourdain, V. Voltage-activated transport of ions through single-walled carbon nanotubes. *Nanoscale* **2017**, *9 (33)*, 11976-11986. https://doi.org/10.1039/c7nr02976d.

3. Lebedev, D.; Malyshev, G.; Ryzhkov, I.; Mozharov, A.; Shugurov, K.; Sharov, V.; Panov, M.; Tumkin, I.; Afonicheva, P.; Evstrapov, A.; Bukatin, A.; Mukhin, I. Focused ion beam milling based formation of nanochannels in silicon-glass microfluidic chips for the study of ion transport. *Microfluid. Nanofluid.* **2021**, *25*, 51. https://doi.org/10.1007/s10404-021-02450-x.
doi.org/10.1007/s10404-021-02450-x

4. Kowalczyk, S.W.; Grosberg, A.Y.; Rabin, Y.; Dekker, C. Modeling the conductance and DNA blockade of solid-state nanopores. *Nanotechnology* **2011**, *22 (31)*, 315101. doi.org/10.1088/0957-4484/22/31/315101.
